# The Role of Preoperative Weight Loss Interventions on Long-Term Bariatric Surgery Outcomes: A Systematic Review

**DOI:** 10.3390/jcm14093147

**Published:** 2025-05-01

**Authors:** Emma MacVicar, James Lucocq, Georgios Geropoulos, Peter J. Lamb, Andrew G. Robertson

**Affiliations:** 1 General Surgery, Aberdeen Royal Infirmary, Foresterhill Health Campus, Aberdeen AB25 2ZN, UK; emma.macvicar2@nhs.scot; 2 Upper Gastro-Intestinal Surgical Unit, Royal Infirmary Edinburgh, 51 Little France Crescent, Edinburgh EH16 4SA, UK; james.lucocq@nhs.scot (J.L.); georgios.geropoulos@nhs.net (G.G.); peter.lamb@nhs.scot (P.J.L.)

**Keywords:** bariatric surgery, pre-operative weight loss, pre-operative optimisation, pre-operative lifestyle interventions, long term outcomes

## Abstract

**Background/Objectives**: The percentage of the world’s population with Class II obesity (body mass index (BMI) ≥ 35) and above is increasing annually. Bariatric (elective weight-loss) surgery is performed for less than one percent of eligible patients. A recent Delphi was unable to reach a consensus recommendation for or against mandated weight loss targets prior to bariatric surgery. This systematic review, performed according to the PRISMA 2020 guidelines, looks at the literature to determine whether there is evidence that pre-operative weight loss affects long-term (≥5 years) outcomes of bariatric surgery (weight loss, co-morbidity resolution). **Methods**: MEDLINE, EMBASE, CABI Digital Library, and Cochrane Central Register of Controlled Trials (Central) were searched from 1 January 2000 to 1 March 2025. Twenty-one full papers were then assessed, and only three papers met the eligibility criteria for inclusion in this review. **Results**: 1072 patients were included (age range 26–73 years, sleeve: bypass 60.3%:49.7%, F:M 70.2%:29.8%). The studies differed in their pre-operative preparation and selection criteria for surgery: one paper used an intensive pre-operative information course and mandated 5% weight loss. This study reported a significant association between pre-operative weight loss and long-term outcomes. Two papers had no mandated pre-operative weight-loss target and found there was no association between pre-operative weight loss and long-term outcomes. **Conclusions**: There is limited and conflicting evidence that pre-operative weight loss percentage affects long-term outcomes of bariatric surgery. Further research looking at five-, ten-, and twenty-year outcomes for bariatric patients is required, particularly randomised controlled trials or tier one evidence.

## 1. Introduction

Obesity is a global health catastrophe, and the number of people affected is increasing annually [1,2]. The World Obesity Federation’s 2025 Atlas predicts that the total number of overweight and obese adults (BMI ≥ 25) will increase by more than 115% 2010–2030 from 524 million to 1.13 billion, with 141 million people (5% of the world’s population) in the Class 2 or 3 (BMI ≥ 35) Obesity Category by 2030 [2]. Currently less than one percent of patients eligible for bariatric surgery undergo an operation [3]. The most recent International Federation for Surgery for Obesity and Metabolic Management in 2023 reported 480,970 bariatric operations were performed in national or regional metabolic bariatric surgery registries, accounting for 81.4% of the known registries worldwide [4]. The predominant primary bariatric operation performed is the Sleeve Gastrectomy (63.3%): Roux-en-Y Gastric Bypass made up 28.8% of primary operations, and other procedures made up 9.9% (including one anastomosis gastric bypass surgery, single-anastomosis duodenoileal bypass with sleeve gastrectomy, biliopancreatic diversion, and adjustable gastric banding). Roux-en-Y Gastric Bypass was the most common revisional bariatric operation. The majority of bariatric patients operated on are female, and the average BMI is reported as 40–45 kg/m^2^ at the time of surgery. Currently the United States of America (USA) performs the largest number of primary bariatric surgeries annually—204,092 recorded in 2023; in comparison, in the United Kingdom (UK), 6118 operations were carried out during that time [4].

Pre-operative preparation prior to bariatric surgery varies greatly within countries and across the world [5,6]. Recently an International Delphi Consensus on patient preparation for metabolic and bariatric surgery was created to help standardize preparation for bariatric surgery. The Delphi included forty-five international clinic experts, and consensus was reached on 90 out of 169 statements (53.3%), including the importance of patient selection criteria, preoperative testing, and the multidisciplinary bariatric team. Consensus was reached that patients should be encouraged to maintain or lose weight prior to surgery; however, no agreement on the benefits or need for pre-operative weight loss targets was made [7]. Weight loss pre-operatively can be a marker of lifestyle change and dietary and behavioral modification. Those supporting pre-operative targets feel it is a marker of commitment, reduces perioperative complications, and improves long-term outcomes. Those opposed to targets feel these are a barrier to progress to surgery [8]. Current enhanced recovery after surgery (ERAS) guidelines 2021 advise that ‘Preoperative weight loss using a very low- or low-calorie diet prior to bariatric surgery should be recommended’, classing the evidence as ‘strong’ [9]. The pre-operative screening process and psychological assessment for patients prior to bariatric surgery are extensive, and many bariatric units mandate enrollment in an exercise programme and/or a mandatory five to ten percent body weight loss prior to listing for bariatric surgery. Insurance companies may also require that patients participate in a six-month supervised weight loss programme prior to funding surgery [10].

The Swedish Obesity Study looked at a large number of patients—22,327—with a median pre-operative weight reduction from 120 to 115 kg (4.8% total weight loss). This study found that the overall risk of complications was reduced by 13% for those with the most pre-operative weight loss (75th percentile—9.5% total weight loss) vs. those patients who had lost the least (25th percentile). For specific complications—anastomotic leakage reduced by 24%; deep infection and abscess by 37%; and minor wound complications by 54% [11]. There is therefore clear evidence that losing weight prior to surgery makes the operation safer in the immediate peri-operative period. A study involving 40,844 patients found that if an anastomotic leak occurred following Roux-en-Y Gastric Bypass, 85% of patients required re-operative surgery, and there was a high requirement for intensive care input: 1 in 4 patients required intensive care, 1 in 25 developed multi-organ failure, and there was a 1 in 100 mortality rate. Complications also significantly delay discharge from the hospital, with the median duration of stay following anastomotic leak being twenty-two days vs. two days for bariatric patients with uncomplicated surgery [12].

There is therefore evidence from the literature that weight loss prior to surgery improves the safety of the operation, decreases complications, and may give an indication as to the commitment the patient has to the process of changing their diet and lifestyle, i.e., affects the peri-operative and short-term outcomes [13]. Studies have also shown that bariatric surgery has a significant positive effect on long-term outcomes: patients have been shown to lose up to 47% excess weight following laparoscopic gastric sleeve vs. 55% excess weight following Roux-en-Y gastric bypass [14].

Some of the most important benefits of this weight loss post-operatively are an improvement in major adverse cardiac events (MACE)—i.e., cardiovascular disease including myocardial infarction, stroke, and death [15,16,17,18]. In addition, bariatric surgery may result in the complete resolution or significant improvement of many co-morbidities caused by obesity—including type two diabetes mellitus; obstructive sleep apnea; hypertension; hyperlipidaemia; gastroesophageal reflux; and idiopathic intracranial hypertension [15,16,17,18,19]. In some cases it has also been shown to have a positive psychological impact, with decreased depression and anxiety, although there is conflicting evidence in the literature [17,18,19]. The question of whether the amount of weight lost pre-operatively has an impact on the long-term (five to ten years and beyond) outcomes for bariatric patients remains to be answered.

This systematic review therefore looks at the literature to determine whether there is evidence that pre-operative weight loss interventions affect the long-term outcomes of bariatric surgery.

## 2. Materials and Methods

### 2.1. Study Design

This review was performed according to the ‘Preferred Reporting Items for Systematic Reviews and Meta-analysis’ (PRISMA) 2020 statement [20] to assess the role of preoperative weight loss interventions on long-term bariatric surgery outcomes. ‘Long term’ was defined as greater than or equal to five years of follow-up. The study protocol was registered with PROSPERO (the University of York Centre for Reviews and Dissemination International Prospective Register of Systematic Reviews), 2025 (Registration number: CRD1005433). Ethical approval was not required as no new data were collected.

### 2.2. Search Strategy

MEDLINE (via PubMed), EMBASE, CABI digital library, and Cochrane Central Register of Controlled Trials (Central) were searched between 1 January 2000 and 1 March 2025. The following search strategy (conducted by authors E.M. and J.L.) was used: [(‘pre-operative weight loss’ or ‘pre-operative lifestyle intervention’ or ‘pre-operative optimisation’) AND (‘bariatric surgery’ or ‘weight loss surgery’ or ‘obesity surgery’ or ‘sleeve gastrectomy’ or ‘gastric bypass’ or ‘Roux-en-Y’) AND (‘long term outcome(s)’)]. Due to the lack of randomised controlled trials, all relevant systematic reviews, meta-analyses, and research papers that fit the inclusion criteria were included.

### 2.3. Review Strategy

Papers were uploaded to and reviewed using Rayyan software 2016 [21]. Duplicate references were removed; the remaining references were then assessed by two blinded reviewers (E.M. and J.L.), and any disagreements were settled by a third independent reviewer (A.R.).

Selected references were reviewed at the full-paper stage, and those meeting Population, Intervention, Control, Outcome, and Study (PICOS) eligibility criteria for inclusion in this review were analysed (Table 1). The population inclusion criteria were studies including only adult patients (≥18 years old) undergoing elective bariatric surgery; emergency surgery and surgery for other reasons were excluded. The intervention of interest was pre-operative weight loss, and the primary outcome was long-term (≥5 years) weight loss following bariatric surgery. The secondary outcome was resolution of co-morbidities, including type two diabetes mellitus, obstructive sleep apnea, and hypertension, and any other secondary outcomes mentioned in the included studies. Non-English language studies were excluded. After selection, the included papers were then read in full by two individual researchers (E.M. and J.L.) who independently extracted the information from each study.

### 2.4. Statistical Analysis and Quality Assessment

Data were collated using Microsoft Excel. We attempted to conduct further statistical analysis, including meta-analysis, but were unable to do so due to the high heterogeneity across the included studies. The Joanna Briggs Institute Checklist for Analytical Cross-Sectional Studies was used to ensure all studies were of high enough quality for inclusion in this review [22]. To assess risk of bias The National Heart, Lung, and Blood Institute (NHLBI) Risk of Bias Tool was used [23]. The included studies were assessed in twelve criteria: (i) study objectives, (ii, iii) study population, (iv) eligible participants, (v) sample, (vi) intervention assessment, (vii) outcome measures, (viii) assessor blinding, (ix) loss to follow-up, and (x, xi, xii) statistical methods/ analysis. Each criterion was rated as “Yes”, “No”, and an overall rating was given (good, fair, or poor) (Appendix A).

## 3. Results

### 3.1. Studies

**Figure 1:**
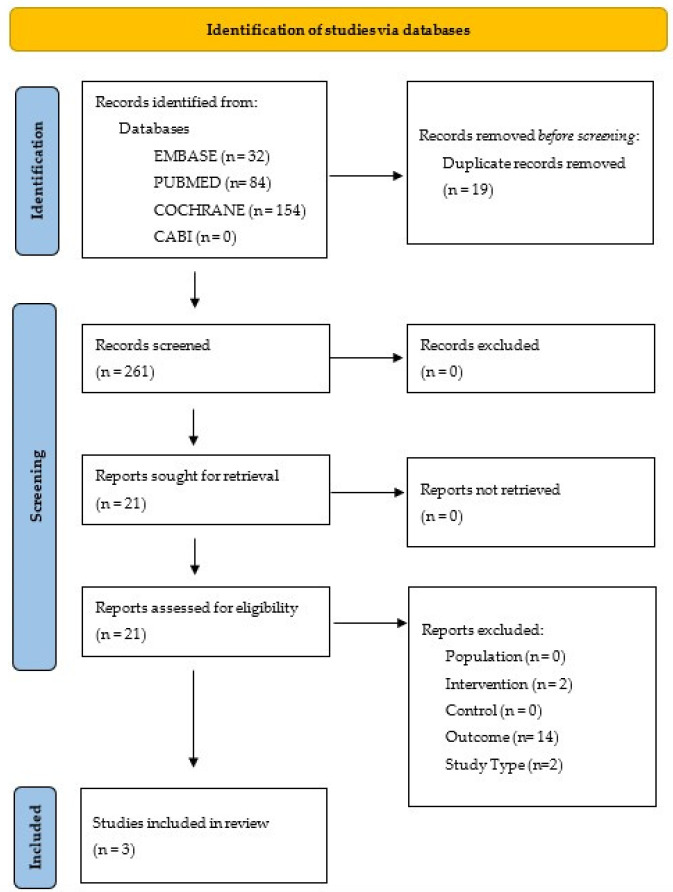
PRISMA Chart.

Two hundred and eighty references were found in total. Nineteen duplicate references were removed; the remaining two hundred and sixty-one references were then assessed by two blinded reviewers, and any disagreements were settled by a third independent reviewer. Twenty-one references were reviewed at the full paper stage, and from this, only three met the PICOS eligibility criteria (Table 1) for inclusion in this review (Figure 1).

There were no randomised controlled trials found in our literature search that met the inclusion criteria. All three studies were retrospective reviews [8,24,25]. Papers were published in 2020, 2021, and 2024, with bariatric operations carried out from 2003 to 2022. All three papers were conducted in single tertiary centres, and all included operations by more than one bariatric surgeon.

Patient demographics are summarized in Table 2. A total of 1072 patients were included across these three papers: Lucocq et al. included 339, Samaan et al. 426, and Tan et al. 306. There were some differences noted between the cohorts: Lucocq et al. was a UK population: Samaan et al. American, and Tan et al. primarily Asian (Chinese, Malay, and Indian). Body mass index on enrollment was similar between cohorts’ range of 42.3–47.3 kg/m^2^.

Co-morbidities across all three populations were similar—including diabetes mellitus; hypertension; obstructive sleep apnea; and hyperlipidemia. All studies included a similar percentage of female vs. male patients (percentage female—75.3% Lucocq et al.; 73.2% Samaan et al.; and 62.1% Tan et al.). The average age of patients was comparable between studies—40.5 years; 45.6 years; and 49 years. Two studies did not include the age range of their patients; the one study that did (Lucocq et al.) had a range of ages from 26 to 73 years. There were some differences between the number of gastric sleeve vs. gastric bypass operations performed. Lucocq et al. included 158 (47.5%) gastric bypass operations and 161 (46.6%) gastric sleeve operations; Samaan et al. included 260 (61%) gastric sleeve operations and 166 (39%) gastric bypass operations; and Tan et al. included 228 (74.5%) gastric sleeve operations and 78 (25.5%) gastric bypass operations.

Each study used a different pre-operative intervention protocol (Table 3). Lucocq et al. used a twelve-week intensive pre-operative information course (IPIC) and mandated a five percent weight loss prior to surgery. Samaan et al. used a pre-operative bariatric programme with an average length of 32.7 weeks and required no set weight loss prior to surgery. Tan et al. prescribed a very low-calorie diet ≤ 800 calories/day (VLCD) using meal replacements for fourteen days preoperatively, and ‘patients were given appropriate nutrition education and individualised diet plans in the weeks-months prior to surgery’; in addition, thorough dietary histories were obtained. In this study no mandated weight loss was required.

One paper—Lucocq et al.—reports that the only variable on seven-year follow-up associated with sustained excess weight loss ≥70% was high pre-operative weight loss (>10.5%) [24]. This study also found that patients who received IPIC had higher rates of achieving excess weight loss ≥50% and ≥70% during long-term follow-up. In contrast to this, Tan et al. found that preoperative weight loss had no significant effect on long-term total weight loss or percentage body mass index lost when measured beyond six months [22]. Samaan et al. also found that pre-operative weight loss was not predictive of postoperative weight loss success after bariatric surgery [8].

### 3.2. Secondary Outcomes

Tan et al. included percentage change in fat mass and waist-hip circumference ratio as secondary outcomes: this study found no significant difference in the effect of pre-operative weight loss on these markers or progress [25]. The other two papers included did not report secondary outcomes.

### 3.3. Quality Assessment

All studies met the Joanne Briggs Institute Checklist for Analytical Cross-Sectional Studies requirements for inclusion in this review. Each study clearly defined their criteria for inclusion in the study, all three papers described the population in detail, and they measured the intervention and outcomes in a reliable way. Confounding factors were considered, and appropriate statistical analysis was used—all studies performed multivariable statistical analysis. Lucocq et al. identified variables significantly associated with excess weight loss using a Kaplan-Meier (KN) analysis, which were then further analysed using the Cox Proportional Hazards Model. Samaan et al. also used ‘multivariable logistical regression models’. Tan et al. used ‘generalised mixed models’ and comparisons calculated using ‘predictive margins from the post-estimation coefficients’: their predictive multivariable model was used for percentage excess body weight loss at one year only.

Bias assessment (Table A1, Appendix A) determined that one study was rated good quality, one fair, and the third poor quality (Table A1). All studies clearly stated the study question, participants were representative of the population of interest (bariatric surgical patients), and intervention and outcomes were fully described and consistently applied to all patients. In addition, all studies used the same pre-operative weight loss programmes for all patients in the study, outcomes were measured consistently for all patients, and appropriate statistics were used. The main area of weakness was blinding: none of the studies used blinding of assessors carrying out analysis. In two of the studies it is difficult to assess if all eligible participants that met the pre-specified criteria were enrolled or not, and none of the studies used an interrupted-time-series design. Tan et al. had a significant loss to follow-up, with 90.2% of their cohort not followed up to the five-year mark, making it significantly less useful in assessing the effects of pre-operative weight loss on long-term outcomes of bariatric surgery.

## 4. Discussion

The evidence for the long-term effect of pre-operative weight loss and outcomes from bariatric surgery is limited; most of the studies found look at the effect of pre-operative weight loss with three years of follow-up or less [26,27,28,29]. We found only three studies that specifically look at pre-operative weight loss with long-term follow-up (≥5 years).

A recent Delphi was unable to reach a consensus decision on the benefits of pre-operative weight loss targets for bariatric surgery, highlighting that there is significant variation in pre-operative preparation for bariatric surgery around the world and limited evidence [5,6,7]. It is interesting that this is reflected within the three papers included in this review, as different conclusions as to the benefit of pre-operative weight loss on long-term outcomes were found. One paper reported a significant difference in long-term weight loss with the implementation of their intensive pre-operative information course (IPIC), which aimed to optimise pre-operative weight loss and provide education prior to bariatric surgery [24]. IPIC involves intensive counselling, psychology, and lifestyle intervention. In contrast, the two other studies showed no association between pre-operative weight loss and long-term outcomes, although both gave patients some form of ‘intensive’ pre-operative intervention: Sammann et al. provided 37.5 weeks of a ‘bariatric programme’. Tan et al. mandated input by ‘endocrinologists, bariatric surgeons, dieticians, bariatric nurses, clinical coordinators, exercise physiotherapists, and psychologists’ in the weeks-months prior to surgery: the exact duration is not specified, and it is unclear if it was standardised for their whole patient cohort [25].

We should therefore consider the question of whether it is in fact the quality of the weight-loss education and intensive counselling that patients received prior to bariatric surgery that played the biggest role in motivating and encouraging people to maintain their long-term weight loss rather than the amount of weight lost pre-operatively alone. In addition, because of the intensive nature of the course and mandated 5% weight loss, it is likely that the patient selection criteria by Lucocq et al. included more motivated patients overall vs. the other studies that required no specific weight loss target pre-operatively. Future studies assessing the impact of pre-operative education and patient motivation vs. weight loss alone are essential to determine the most effective pre-operative protocol for bariatric patients. In addition, larger collaborative studies between different bariatric centres around the world are needed to investigate individual pre-operative protocols and determine which is the most effective for our patients.

Only 1072 patients in total were included in these three papers; however, Tan et al. had a significant number of patients that were not reviewed at their longest—5-year review point: only 30 patients were reviewed here from a total of 306 included in the study, i.e., 90.2% of the patients were not reviewed at the 5-year mark [25]. This significantly limits the usefulness of this study in assessing the role of pre-operative weight loss and long-term outcomes. Lucocq et al. had a median follow-up time of 7 years, IQR range 4–9, range 0.5–11 years [24], and Samaan et al. had an ‘average follow-up of 6.3 years’ [8]. Therefore, from the 1072 total patients, we can gather that the maximum number of patients reviewed at the five-year point is 795. The actual number is lower than this due to the range in follow-up times reported for each study; this highlights the limited data that is available regarding pre-operative weight loss and long-term outcomes for patients following bariatric surgery.

There were also some differences in the type of operation performed: Lucocq et al. included 47.5% gastric bypass operations, vs. 39% for Samaan et al. and 25.5% for Tan et al. Lucocq et al. comment from their own findings that ‘sleeve gastrectomy was also associated with more unsustained excess weight loss in comparison to gastric bypass’. A 2024 Lancet study has shown that Roux-en-Y gastric bypass gives a significantly higher total amount of weight loss and improved secondary outcomes—including decreased dyslipideamia and gastroesophageal reflux—the caveat being that they found a higher rate of minor complications associated [14]. The difference in operations performed between the three studies may partly contribute to why they had different conclusions. There has been huge growth and change within bariatric surgery since the first introduction of open intestinal bypass procedures in the mid-1950s. Gastric bypass surgery was introduced in 1967, and at that time there were no guidelines for patient selection or choice of operation; therefore, each surgeon practiced based on their own experience and preferences [4]. The progression from open to laparoscopic surgery as standard and now the increasing use of robotic surgery again highlights the progression and expansion in this area of surgery. We are also seeing increased revisional operations and the introduction of novel procedures. For example, the single-anastomosis duodeno-ileostomy with sleeve (SADI-S) and the one-anastomosis gastric bypass (OAGB) have only recently been endorsed by the American Society for Metabolic and Bariatric Surgery (ASMBS) despite having been performed for some time. New technology is also playing a role, one example being the use of the magnet system for duodeno-ileostomy (MSDI) to increase precision and decrease complications [30]. Future studies looking at both the short- and long-term outcomes of these procedures and new technology are essential so that the most effective and safest procedures can be carried out for bariatric patients.

One of the problems with the use of weight loss targets for bariatric surgery is the fact that it introduces delays and barriers for patients. Currently, up to 60% of patients initially inquiring about bariatric surgery have been found to drop out prior to surgery. One of the main reasons for attrition given by patients in qualitative semi-structured interviews was the burden of pre-operative workup requirements, alongside stigma, fear of surgery, and anticipated regret [31]. The Swedish Obesity Study (SOS) trial showed that a higher starting BMI prior to bariatric surgery was predictive of poorer long-term outcomes [11]. Lucocq et al. found the same result in a UK cohort: those with higher starting BMI had worse outcomes at five and seven years. They also showed that higher age negatively affected excess weight loss (≥50%) following laparoscopic sleeve gastrectomy [24]. Bariatric surgery is a limited and important resource: perhaps instead of targets and a defined amount of pre-operative weight loss that introduce delays and barriers for patients seeking help, earlier intervention needs to be the focus to prevent people from reaching the extremes of BMI where lifestyle and dietary habits may become so entrenched that making changes without pharmaceutical or surgical assistance becomes extremely challenging if not impossible for a large number of individuals.

A modified Association of Upper Gastro-Intestinal Surgeons (AUGIS) Delphi Consensus on the most important research topics in bariatric and metabolic surgery (2019) highlighted eleven key research topics—these included research to elicit greater understanding of the pathophysiology of the different procedures and also the ‘long-term sequelae’ to bariatric and metabolic surgery [32]. They also highlighted the importance of determining the best practice for deciding which patients would have the greatest benefit from surgery, which is particularly relevant in the United Kingdom, where bariatric surgery is delivered free at the point of service via the National Health Service (NHS). Further research into the effect of pre-operative weight loss, including qualitative studies with input from patients, including those who opt to go ahead with surgery and those who do not, is required to reach these aims, and research into the effects of pre-operative weight loss will add to our understanding in this area.

It is interesting that there is little discussion of secondary outcomes within the three papers that met the inclusion criteria for this review—one paper used percentage change in fat mass and change in waist/hip circumference ratio; none looked at comorbidity resolution. A recent 2025 study has developed criteria for measuring and recording the long-term outcomes of bariatric surgery [33]—the use of standardised outcome measures will aid future systematic reviews; improve the heterogeneity between studies to facilitate meta-analysis and more accurate comparison and analysis of the data available. In addition, it is important to consider the quality of the studies—only one was rated as high quality; one moderate; and one poor based on the National Heart; Lung; and Blood Institute (NHLBI) Risk of Bias tool. The quality of evidence is important as it reflects the extent to which confidence in an estimate of the effect is adequate to support a particular recommendation, in this case pre-operative weight loss and long-term outcomes of bariatric surgery. The two studies rated as moderate and poor both showed no association between pre-operative weight loss and long-term weight loss, vs. the one study rated as high quality did show an association. This imbalance in study quality may have impacted the overall findings and interpretation of results and highlights the need for further well-designed, unbiased studies in bariatric surgery.

The research studies in this review were carried out prior to the recent widespread use of Glucagon-like Peptide 1 agonist (GLP-1) and Glucose-Dependent Insulinotropic Polypeptide (GIP) receptor agonist medications by bariatric patients for weight loss, and therefore the pre-operative weight loss reported likely represents behavioral modification secondary to dietetic and psychological input. There is evidence that participation in pre-operative education influences the motivation of people to make the lifestyle changes required for successful, sustained weight loss post-bariatric surgery [34,35,36]. How big a role percentage weight loss prior to surgery plays in the long term remains unclear, although it may be a surrogate marker for lifestyle change and behavioral modification. The increase in popularity of medications worldwide may alter the effects of weight loss pre-operatively in the long term. Future studies in this area should therefore consider the role of pharmacological interventions when examining pre-operative intervention strategies.

## 5. Strengths and Limitations

This is the first systematic review looking at the evidence for pre-operative weight loss in long-term outcomes following bariatric surgery. This study’s main limitation is the lack of published literature available. The absence of randomised controlled trials and tier one evidence also limits our ability to draw definite conclusions. Every attempt has been made to reduce bias by ensuring reviewers were blinded to the other reviewers’ decisions and able to give their own independent assessment of which papers met the inclusion criteria.

## 6. Conclusions

At present there is limited evidence describing the impact of pre-operative weight loss on the long-term outcomes of bariatric surgery; however, it may represent a surrogate marker of positive lifestyle change and behavioral modification, which should be encouraged. Further studies looking at the long-term—five; ten; twenty-year—outcomes of pre-operative weight loss on bariatric surgery are required to allow bariatric teams to make the best possible decisions for those seeking weight-loss surgery and optimise the long-term outcomes of surgery for patients. The question of whether mandated pre-operative weight loss targets are beneficial in the long term needs to be researched in more detail.

## Figures and Tables

**Table 1:** Inclusion/ Exclusion Criteria.

	Inclusion	Exclusion
Population	Adult Population ≥ 18 years old Elective Operations Bariatric Surgery	Paediatrics/Children < 18 years old Emergency Operations Non-Bariatric Surgery
Intervention	Pre-operative Weight Loss	Studies Not Including Pre-operative Weight Loss
Control	Ideally control group present	
Outcome	Long term (≥5 year Post-operative) Weight Loss Long-term (≥5 years Postoperative) Resolution of Co-morbidities	Reports Short-Term Outcomes Only <5 Years Follow-up
Study	All Study Types Included Human Studies English Language	Animal Studies Non-English Language Studies

**Table 2:** Summary of Population Demographics: Study Comparison.

	Lucocq et al. [24]	Samaan et al. [8]	Tan et al. [25]
Total Number Patients	339	426	306
Average age (years)	49 (median)	45.6 (mean)	40.5 (mean)
Age range (years)	26–73	Not recorded	Not recorded
% Female	75.3%	73.2%	62.1%
Enrollment BMI (kg/m^2^)	47.2 (median)	47.3 (mean)	42.3 (mean)
Operation: Sleeve	158 (46.6%)	260 (61%)	228 (74.5%)
Bypass	161 (47.5%)	166 (39%)	78 (25.5%)
Co-morbidities:			
Diabetes mellitus	129 (38.1%)	125 (29.3%)	118 (41.7%)
Hyperlipidaemia	Not recorded	139 (32.6%)	120 (39.2%)
Hypertension	96 (28.3%)	232 (54.5%)	153 (50.0%)
Sleep apnoea	36 (10.6%)	154 (36.2%)	Not recorded
Psychiatric	150 (44.2%)	Not recorded	Not recorded
GERD	Not recorded	207 (48.5%)	Not recorded

**Table 3:** Study Comparison: Intervention, Primary and Secondary Outcomes.

	Lucocq et al. [24]	Samaan et al. [8]	Tan et al. [25]
Follow-up Time	7 years (median)	6.3 years (mean)	5 years (maximum)
Intervention	12 week intensive IPIC Mandated 5% weight loss	Pre-operative bariatric programme (mean 32.7 weeks) No mandated weight loss	14 days preoperative Very low-calorific diet ≤800 calories/day No mandated weight loss
Comparison groups— by pre-operative weight loss %	Excess weight loss <10.5% Excess weight loss ≥10.5%	Excess weight loss <5%: (30%) Excess weight loss ≥5, <10%: (16%) Excess weight loss ≥10%: (54%)	Lost <5% Body weight pre-op: n = 267 (87.3%) Lost ≥5% Body weight pre-op: n = 39 (12.7%)
Primary Outcomes	Excess weight loss <50% Excess weight loss ≥50% Excess weight loss ≥70%	Excess weight loss % BMI points lost	% Total Weight Loss % Excess Body Mass Index Loss
Secondary Outcomes (if recorded)	-	-	% Change in fat mass Waist-hip circumference ratio

## Data Availability

No new data were created or analysed in this study. Data sharing is not applicable to this article.

## Abbreviations

The following abbreviations are used in this manuscript:

IPIC	Intensive pre-operative information course
PRISMA	Preferred Reporting Items for Systematic Reviews and Meta-analysis
PICOS	Population, Intervention, Comparison, Outcome, Study
GLP-1	Glucagon-like peptide-1

## Appendix A

**Table A1:** Quality Assessment.

First Author	Year Published	Q1	Q2	Q3	Q4	Q5	Q6	Q7	Q8	Q9	Q10	Q11	Q12	Total	Quality Score
														Score	
Lucocq [24]	2024	Y	Y	Y	Y	Y	Y	Y	N	Y	Y	N	NA	9	Good
Samaan [8]	2021	Y	Y	Y	CD	Y	Y	Y	N	Y	Y	N	NA	8	Fair
Tang [25]	2020	Y	Y	Y	CD	N	Y	Y	N	N	Y	N	NA	6	Poor

Table A1: Key Y = Yes. N = No. Not applicable = NA. CD, cannot determine. 1. Was the study question or objective clearly stated? 2. Were eligibility/selection criteria for the study population pre-specified and clearly described? 3. Were the participants in the study representative of those who would be eligible for the test/service/intervention in the general or clinical population of interest? 4. Were all eligible participants that met the pre-specified entry criteria enrolled? 5. Was the sample size sufficiently large to provide confidence in the findings? 6. Was the test/service/intervention clearly described and delivered consistently across the study population? 7. Were the outcome measures pre-specified, clearly defined, valid, reliable, and assessed consistently across all study participants? 8. Were the people assessing the outcomes blinded to the participants’ exposures/interventions? 9. Was the loss to follow-up after baseline 20% or less? Were those lost to follow-up accounted for in the analysis? 10. Did the statistical methods examine changes in outcome measures from before to after the intervention? Were statistical tests conducted that provided p-values for the pre-to-post changes? 11. Were outcome measures of interest taken multiple times before the intervention and multiple times after the intervention (i.e., did they use an interrupted time-series design)? 12. If the intervention was conducted at a group level (e.g., a whole hospital, a community, etc.) did the statistical analysis take into account the use of individual-level data to determine effects at the group level?
